# Small molecule epigenetic screen identifies novel EZH2 and HDAC inhibitors that target glioblastoma brain tumor-initiating cells

**DOI:** 10.18632/oncotarget.10661

**Published:** 2016-07-18

**Authors:** Natalie Grinshtein, Constanza C. Rioseco, Richard Marcellus, David Uehling, Ahmed Aman, Xueqing Lun, Osamu Muto, Lauren Podmore, Jake Lever, Yaoqing Shen, Michael D. Blough, Greg J. Cairncross, Stephen M. Robbins, Steven J. Jones, Marco A. Marra, Rima Al-Awar, Donna L. Senger, David R. Kaplan

**Affiliations:** ^1^ Program in Neurosciences and Mental Health, The Hospital for Sick Children, Toronto, Canada; ^2^ Drug Discovery Group, Ontario Institute for Cancer Research, Toronto, ON, Canada; ^3^ Arnie Charbonneau Cancer Institute, Department of Oncology, Cumming School of Medicine, University of Calgary, Calgary, Alberta, Canada; ^4^ Canada's Michael Smith Genome Sciences Centre, BC Cancer Agency, Vancouver, Canada; ^5^ Department of Medical Genetics, University of British Columbia, Vancouver, British Columbia, Canada; ^6^ Department of Molecular Biology and Biochemistry, Simon Fraser University, Burnaby, British Columbia; ^7^ Department of Molecular Genetics, University of Toronto, Toronto, ON Canada

**Keywords:** glioblastoma, drug discovery, epigenetics, UNC1999, HDAC inhibitor

## Abstract

Glioblastoma (GBM) is the most lethal and aggressive adult brain tumor, requiring the development of efficacious therapeutics. Towards this goal, we screened five genetically distinct patient-derived brain-tumor initiating cell lines (BTIC) with a unique collection of small molecule epigenetic modulators from the Structural Genomics Consortium (SGC). We identified multiple hits that inhibited the growth of BTICs *in vitro*, and further evaluated the therapeutic potential of EZH2 and HDAC inhibitors due to the high relevance of these targets for GBM. We found that the novel SAM-competitive EZH2 inhibitor UNC1999 exhibited low micromolar cytotoxicity *in vitro* on a diverse collection of BTIC lines, synergized with dexamethasone (DEX) and suppressed tumor growth *in vivo* in combination with DEX. In addition, a unique brain-penetrant class I HDAC inhibitor exhibited cytotoxicity *in vitro* on a panel of BTIC lines and extended survival in combination with TMZ in an orthotopic BTIC model *in vivo*. Finally, a combination of EZH2 and HDAC inhibitors demonstrated synergy *in vitro* by augmenting apoptosis and increasing DNA damage. Our findings identify key epigenetic modulators in GBM that regulate BTIC growth and survival and highlight promising combination therapies.

## INTRODUCTION

Glioblastoma (GBM) is the most lethal and aggressive adult brain tumor. The standard-of-care treatment includes surgical resection, followed by radiotherapy and chemotherapy with the alkylating agent temozolomide (TMZ). Despite this multifaceted therapy, prognosis for GBM patients remains poor with a median survival of only 14.6 months [1]. There are numerous hurdles impeding the development of successful GBM treatments including high inter- and intra-tumoral heterogeneity [2, 3], the inability of 98% known drugs to cross the blood-brain barrier [4] and the presence of a population of stem cell-like glioma cells, referred to as brain tumor-initiating cells (BTICs), that are thought to contribute to GBM propagation, treatment resistance and tumor recurrence [5–7]. Therefore, to further improve the survival of GBM patients, new therapeutic strategies including combination-based therapies need to be considered.

Epigenetic mechanisms are being increasingly recognized as a major factor contributing to the pathogenesis of GBM [8, 9]. Epigenetic silencing of O-6-methylguanine-DNA methyltransferase (MGMT) by promoter hypermethylation is associated with significantly longer survival [10]. Recent genome-wide genomic and epigenomic analyses have revealed that 46% of 291 GBM samples tested have at least one somatic mutation in genes associated with chromatin modification [11]. Epigenetic alterations, particularly those involving enzymatic modifications of either DNA or associated histone proteins are currently being exploited for therapeutic drug targeting. However, at the moment there is limited knowledge regarding the mechanisms through which epigenetic modifiers function in GBM, and the possibility of therapeutic targeting has not been rigorously tested for this tumor.

In the current study, we performed a drug screen on five genetically-distinct BTIC lines with a unique collection of small molecule epigenetic inhibitors compiled by the Structural Genomics Consortium (SGC), and validated our findings on these and 10 additional lines. The BTIC lines were generated from tumors from newly diagnosed and recurrent GBM patients [12–17], exhibit the ability to self-renew and differentiate into multiple neural cell lineages [14], encompass the diversity of molecular genetic alterations that are known to occur in human GBM patients (e.g. EGFR, PTEN, p53, IDH1 etc.) and are tumorigenic in orthotopic xenograft murine models [14, 18]. We identified multiple hits, and further evaluated the therapeutic potential of compounds regulating two epigenetic modifiers, enhancer of zeste homolog 2 (EZH2) and histone deacetylase (HDAC), both of which have high relevance for GBM.EZH2 as well as HDAC1 and HDAC2 are over-expressed in GBM [19–23] and are associated with shorter overall survival [19, 20, 22]. We show here that the SAM-competitive EZH2 inhibitor UNC1999 exhibits low micromolar cytotoxicity *in vitro* on a diverse collection of BTIC lines, synergizes with Dexamethasone (DEX) and suppresses tumor growth *in vivo* in combination with DEX. Furthermore, we demonstrate that a unique brain-penetrant class I HDAC inhibitor is cytotoxic *in vitro* on a panel of BTIC lines and is able to extend survival in combination with TMZ in an orthotopic model *in vivo*. Finally, we demonstrate that the combination of EZH2 and HDAC inhibitors shows synergy *in vitro* by augmenting apoptosis and increasing DNA damage of GBM tumors.

## RESULTS

### Small molecule screen identifies epigenetic modulators that target diverse BTICs

**Figure 1:**
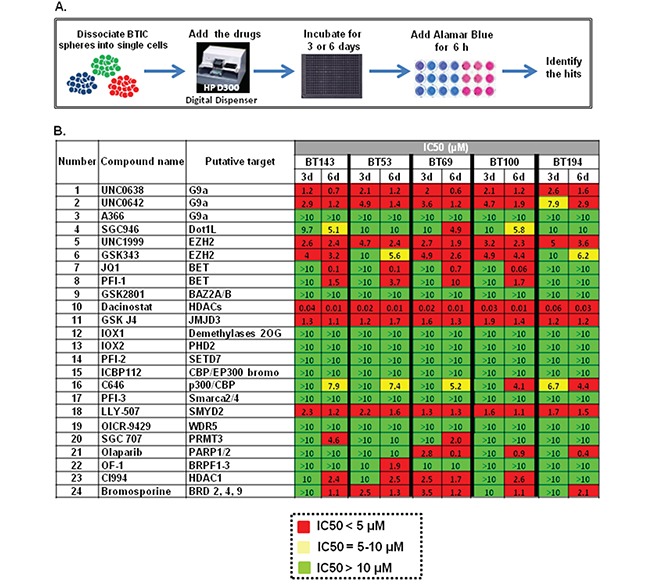
Testing of the SGC epigenetic library of chemical probes on five BTICs lines **A.** Detailed design of the screening procedure; **B.** Epigenetic screen results. Compounds that exhibit IC_50_ < 5 μM were defined as hits and are shown in *red*, drugs with 5 μM < IC_50_ < 10 μM are marked in *yellow* and inactive compounds that exhibit IC_50_ > 10 μM are shown in *green*.

To identify epigenetic modulators that could inhibit the growth of BTICs *in vitro*, we screened five genetically distinct patient-derived BTIC lines with an SGC collection of 24 chemical probes (Figure 1A). Compound addition was performed digitally using 12-point serial dilutions, and alamarBlue reduction was performed after 3 or 6 days to assess metabolic activity as an indicator of cell health. Compounds exhibiting IC_50_ values lower than 5 μM in either the 3 day or a 6-day screening assays were defined as hits (marked in red, Figure 1B). Compounds targeting multiple epigenetic targets were identified that suppressed BTIC health at low micromolar or nanomolar ranges, including histone methyl transferases (G9A, EZH2, SMYD2), BET bromodomains, histone demethylase (JMJD3) and histone deacetylases (HDACs). Compounds that targeted BET ((+)-JQ1) and JMJD3 (GSKJ4) have been recently described as promising compounds for GBM and pediatric GBM [24–26], and therefore we decided to not pursue them further. We tested the compounds targeting G9A and SMYD2 for their putative target inhibition (H3K9me2 and H3K4me1/H3K36me2, respectively) and found them inactive, suggesting that their cytotoxicity could be due to off-target effects. Therefore, we decided to focus on novel compounds targeting EZH2 and HDAC, both of which have high relevance for GBM [19–22, 27].

### Targeting EZH2 in BTICs using a novel SAM-competitive inhibitor UNC1999

**Figure 2:**
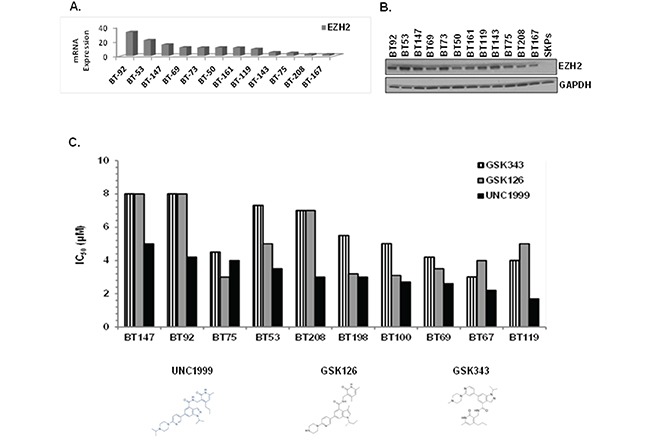
EZH2 is overexpressed in BTICs and SAM-competitive EZH2 inhibitors, in particular UNC1999, are cytotoxic *in vitro* **A.** EZH2 mRNA expression in BTICs. **B.** EZH2 protein expression in BTICs and SKPs by immunoblotting with anti-EZH2 antibody. **C.** IC_50_ values presented as bar graph for SAM-competitive EZH2 inhibitors using alamarBlue as a read-out.

EZH2 is the catalytic subunit of the Polycomb Repressive Complex 2 (PRC2) that inhibits gene expression through tri-methylation of lysine 27 on histone H3 (H3K27me3). Deregulation of EZH2 has been documented in various cancer types, both solid and hematological, and is associated with poor survival [28–30]. Therefore, we first assessed EZH2 expression in a panel of genetically-diverse BTIC lines. We observed EZH2 mRNA (Figure 2A) and protein (Figure 2B) in all BTIC lines tested, with virtually no expression in skin-derived precursor cells (SKPs), which are primary postnatal human neural-like dermal stem cells used as a normal neural stem cell control. While we did not observe in BTIC lines EZH2 activating point mutations Y641 and A677 that occur in hematological malignancies, an increase in copy number variations (CNV) was detected in the majority of those lines (Supplementary Table 1).

We next compared the SAM-competitive EZH2 inhibitors GSK126, GSK343, UNC1999 and EPZ6438 on 10 BTIC lines for their ability to suppress cell health in the alamarBlue assay. UNC1999, a SAM-competitive EZH2/EZH1 inhibitor [31] identified in our screening of the SGC library, exhibited superior ability to suppress cell health as compared to the other inhibitors (IC_50_ = 2-5 μM; Figure 2C), with EPZ6438 being inactive even at 10 μM (data not shown). UNC2400, an inactive analogue of UNC1999 [29, 31], did not affect cell growth at 10 μM or lower, suggesting that the biological effects of UNC1999 were specific to its activity as a H3K27 trimethylation inhibitor. To address why other EZH2 inhibitors did not show similar efficacy to UNC1999, we compared the target inhibition of UNC1999 with three other EZH2 inhibitors, GSK343, GSK126 and EPZ-6438 at the same concentrations (2 μM and 3 μM) on BTICs. We assessed different potential targets including H3K27me3, H3K27me2, H3K9Me2, H3K4Me1 and H3K27ac as well as EZH2 and total histone H3 (Supplementary Figure 1). UNC1999 inhibited only trimethylation of H3K27 without affecting the other targets or altering the total level of EZH2 or Histone H3. The other three inhibitors suppressed trimethylation of H3K27, GSK126 and EPZ-6438 also inhibited dimethylation of H3K27, and GSK343 also reduced H3K9me2 and H3K4me1 levels. Our conclusion is that UNC1999 is more specific than the other inhibitors and therefore, we focused on UNC1999 for our further studies.

### UNC1999 treatment of BTICs decreases cell viability, impairs self-renewal, causes cell cycle arrest and decreases H3K27Me3 levels

**Figure 3:**
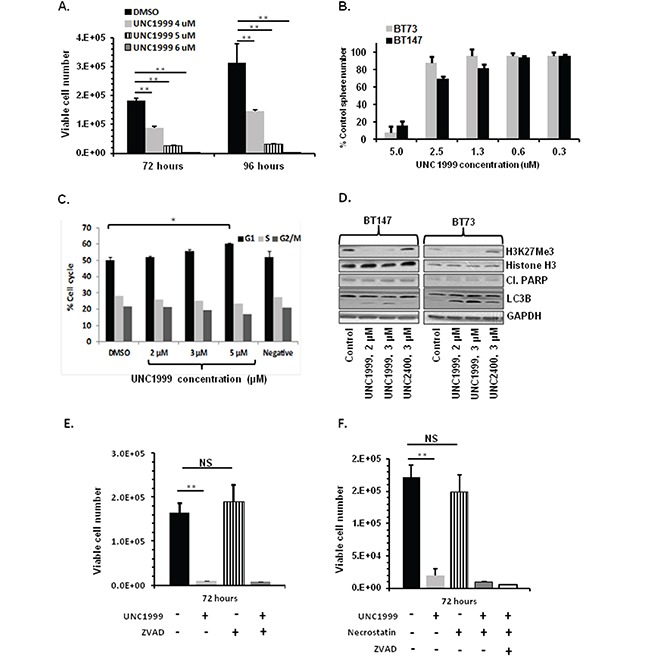
UNC1999 treatment decreases cell viability, impairs self-renewal, causes cell cycle arrest and decreases H3K27Me3 **A.** Treatment with UNC1999 (4-6 μM) reduces viable cell numbers in BT73 as assessed by trypan blue exclusion (n=3; ** P<0.01). **B.** Treatment with varying concentrations of UNC1999 impairs self renewal in BT73 and BT147 as assessed by sphere assay (n=3). **C.** Treatment with UNC1999 (2-5 μM) induces G1 cell cycle arrest in BT73 as assessed by propidium iodide staining. **D.** Representative western blot demonstrates the effect of treatment with UNC1999 and the negative control UNC2400 on H3K27Me3, total levels of Histone H3, cleaved-PARP and LC3B in BT73 and BT147. **E.** Treatment with UNC1999 (5 μM) and ZVAD-FMK (20 μM) for 72 hours does not rescue cell viability in BT73 as assessed by trypan blue exclusion (n=3; ** P<0.01). **F.** Treatment with UNC1999 (5 μM) and Necrostatin (50 μM) for 72 hours does not rescue cell viability in BT73 as assessed by trypan blue exclusion (n=3; ** P<0.01).

We first asked whether treatment of BTICs with UNC1999 affected cell viability. The BT73 line was treated with increasing concentrations of UNC1999 (4-6 μM) and viable cells were counted at 72 and 96 hours post-treatment. UNC1999 significantly decreased viable cell numbers in a dose-dependent manner as compared to DMSO-treated cells at all time points tested (Figure 3A). Next, the effect of UNC1999 on self-renewal was investigated. For this, BT73 and BT147 cells were treated with varying doses of the inhibitor and the number of spheres counted after 6 days. UNC1999 completely abrogated sphere formation at 5 μM in both BTIC lines (Figure 3B). To ask whether UNC1999 also had cytostatic effects on BTIC lines, the percentage of cells in the different stages of the cell cycle was examined by flow cytometry. BT73 cells were treated with varying concentrations of UNC1999 (2-5 μM), and cellular DNA content was examined 48 hours later. The percentage of cells in the G_1_ phase of the cell cycle significantly increased as a consequence of drug treatment, with a slight decrease in the percentage of cells in both S and G_2_/M phases (Figure 3C). In contrast, there were no changes in the percentage of cells at different stages of the cell cycle in cells treated with UNC2400, an inactive analogue of UNC1999 [29, 31]. In BTICs, UNC1999 treatment completely inhibited trimethylation of lysine 27 on Histone H3 (H3K27) without affecting the total levels of either Histone H3 or EZH2 (Figure 3D). Although there were no changes detected in the expression of the apoptotic marker cleaved-PARP as a consequence of drug treatment, we observed an increase in the expression of LC3B II, suggesting that the potential mechanism of cell death could be autophagy rather than apoptosis (Figure 3D), in agreement with the reported autophagic mechanism of GSK343 and UNC1999-mediated cell death in breast and lung carcinoma cell lines [32]. In order to rule out apoptosis and necroptosis, we used Annexin V assay to assess the percentage of apoptotic cells. We could not detect Annexin V-positive cells at either 24, 48 or 72 hours following UNC1999 treatment (data not shown). Moreover, co-incubation of cells with UNC1999 and pan-caspase inhibitor Z-VAD-FMK did not rescue the cells from dying (Figure 3E), suggesting that UNC1999 induces cell death independently of apoptosis. Necroptosis was apparently not involved in UNC1999-induced cell death. Co-incubation of cells with Necrostatin, a necroptosis inhibitor [33], failed to rescue UNC1999-induced cell death (Figure 3F), and co-incubating the cells with UNC1999 and both Necrostatin and Z-VAD-FMK also did not have an effect on cell viability, confirming that these mechanisms are not compensating for each other. Since UNC1999 increased the expression of the autophagic marker LC3B II, we suggest that autophagy rather than necroptosis or apoptosis is the mechanism of BTIC cell death by this compound.

### The combination of UNC1999 and dexamethasone is synergistic *in vitro* and suppresses tumor growth *in vivo*

**Figure 4:**
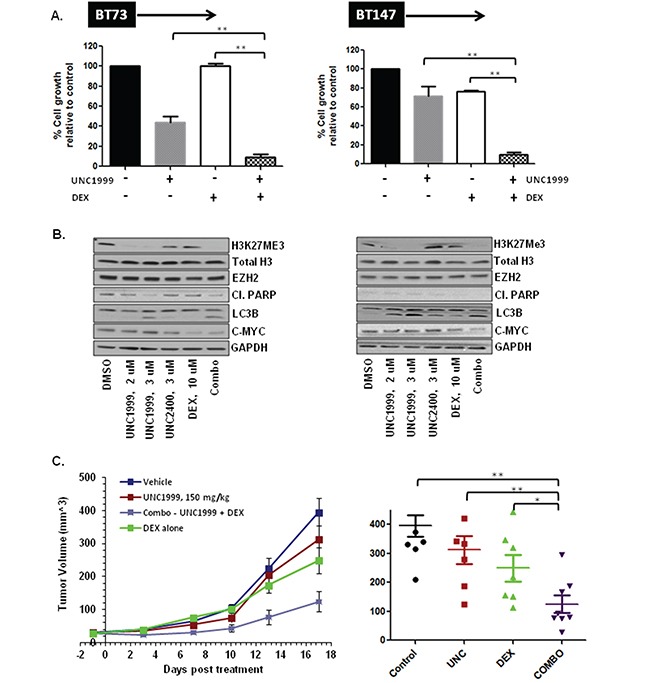
UNC1999 is synergistic with dexamethasone (DEX) *in vitro* and suppresses tumor growth *in vivo* in a flank xenograft model **A.** Representative bar graphs demonstrating synergy between UNC1999, 3.7 μM and DEX, 31 μM *in vitro.* **B.** Representative western blots demonstrating assessment of different targets following treatment with drugs alone and a combination *in vitro.* **C.** NOD/SCID mice bearing ~25 mm^3^ tumors were randomized into 4 groups: 1) vehicle; 2) UNC1999, 150 mg/kg, gavage; 3) DEX, 1 mg/kg, i.p. and 4) combination. Three independent experiments were performed. Representative tumor growth data and tumor volume at 2 weeks after treatment initiation are shown (*p<0.05; ** p<0.01).

We next asked whether UNC1999 efficacy *in vitro* can be augmented through combination with additional drugs. We first tested a combination of UNC1999 and Temozolomide (TMZ), a known GBM chemotherapeutic agent, on two BTIC lines (BT73 and BT147). The results were analyzed using the CalcuSyn median effect model, where the CI indicates synergysm (CI<0.9), additivity (CI=0.9-1.1) and antagonism (CI>1.1). We found that at all concentrations tested there was no synergy or additivity detected in a combination of UNC1999 and TMZ with CI values at the ED_50_= 2.09, ED_75_=1.38 and ED_90_=1.19 in BT73 and ED_50_=1.2, ED_75_=1.19 and ED_90_=1.19 in BT147 (Supplementary Figure 2). We then examined a combination of UNC1999 with Dexamethasone (DEX), a corticosteroid commonly used to treat brain edema in GBM patients. It has been previously shown that a combination of a different EZH2 inhibitor, EPZ-6438, was synergistic with glucocorticoid receptor agonists such as prednisolone and dexamethasone in B cell lymphoma [34]. In agreement with those findings, we found that a combination of UNC1999 with DEX was synergistic in two different BTIC lines *in vitro* with CI values at the ED_50_=0.87, ED_75_=0.82 and ED_90_=0.78 in BT73 and ED_50_=0.84, ED_75_=0.78 and ED_90_=0.73 in BT147 (Figure 4A). There were no additional effects on H3K27me3 levels as a consequence of combination (Figure 4B), nor were there changes in EZH2 protein levels, total Histone H3 or cleaved-PARP. We did observe a decrease in c-MYC protein expression following treatment with the combination of drugs, although DEX alone was also able to suppress c-MYC. Moreover, we observed no additional increase in LC3B II. To investigate further potential autophagy mechanisms, the effect of UNC199 or DEX alone and in combination on p62/SQTM1, a known autophagy substrate that decreases as a consequence of ongoing autophagy, was examined. We found that in both BT73 and BT147 lines, p62 levels increased in the combination group as compared to the DMSO control and with the single drugs alone (Supplementary Figure 3). These findings are similar to other reports showing that impairment of autophagic flux results in autophagy-induced cell death [35, 36].

We next evaluated the efficacy of UNC1999 treatment alone and in combination with DEX *in vivo*. To assess whether UNC1999 crossed the blood brain barrier and accumulated to sufficient concentrations *in vivo*, we performed a pharmacokinetic analysis of plasma and brain samples using LC/MS. Following oral administration of 150 mg/kg, a dose in which UNC1999 elicited no toxicity, the concentration in the plasma was ~7 μM and only 0.5 μM in the brain at both 30 min and 4 hours. Since this brain concentration was 10-fold less than the *in vitro* IC_50_ on the BTIC lines, we did not proceed with an orthotopic model. We instead tested its efficacy in a flank xenograft model where we found that the concentration of the drug in the tumor was ~13 μM, as a proof of concept. For this analysis, NOD/SCID mice with small established BT73 tumors were treated with either vehicle, UNC1999 alone (150 mg/kg), DEX alone (1 mg/kg) or a combination of the two drugs for 17 days. To evaluate target inhibition *in vivo*, we isolated tumors 10 days after initiation of treatment with 150 mg/kg of UNC1999. UNC1999 decreased the trimethylation of H3K27 in a similar fashion to that observed *in vitro* (Supplementary Figure 4), indicating that UNC1999 exhibits potent target inhibition both *in vitro* and *in vivo.* As shown in Figure 4C, UNC1999 alone was ineffective at suppressing tumor growth. Treatment with DEX alone had only a partial effect, while treatment with both UNC1999 and DEX significantly suppressed tumor growth as compared to control or single-agent treatment.

### HDAC inhibitor (compound 26) treatment of BTICs decreases cell viability, impairs self-renewal, causes cell cycle arrest, induces apoptosis and increases acetylation of histone H3

**Figure 5:**
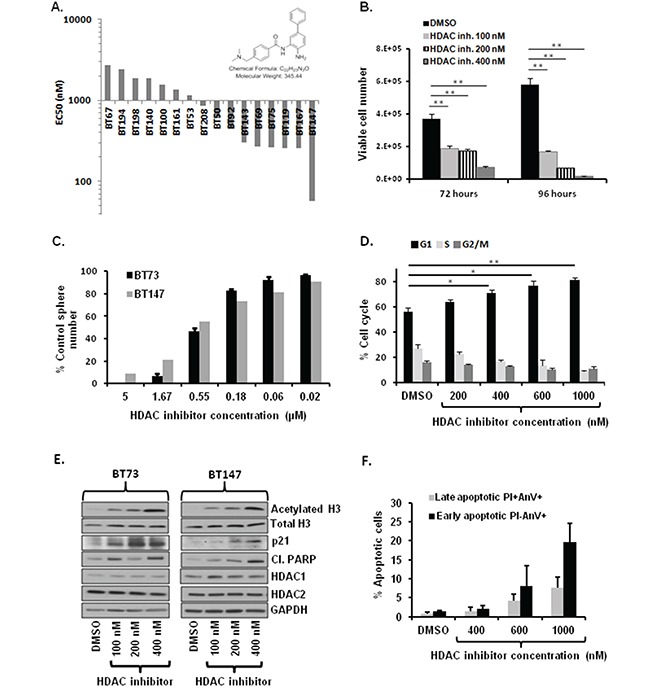
Treatment with brain-penetrant HDAC inhibitor compound 26 decreases cell viability, impairs self-renewal, causes cell cycle arrest, induces apoptosis and increases total levels of acetylated H3 **A.** Compound 26 is cytotoxic on diverse BTIC lines *in vitro*. Waterfall graph with IC_50_ values is shown. **B.** Treatment with compound 26 (100-400 nM) reduces viable cell numbers in BT73 as assessed by trypan blue exclusion (n=3; ** P<0.01). **C.** Treatment with varying concentrations of compound 26 impairs self renewal in BT73 and BT147 as assessed by sphere assay (n=3). **D.** Treatment with compound 26 (200-1000 nM) induces G1 cell cycle arrest in BT73 as assessed via Propidium iodide staining. **E.** Representative western blot demonstrates effect of treatment with compound 26 (100-400 nM) on total acetylation of histone H3, total levels of Histone H3, HDAC1/2, p21 and cleaved PARP in BT73 and BT147. **F.** Treatment with compound 26 (400-1000 nM) increases the percentage of early and late apoptotic cells as assessed via Annexin V staining (n=3).

Two class I HDAC inhibitors were identified as hits from the epigenetic screen, Dacinostat and CI994. Both HDAC1 and HDAC2 mRNA and protein were detected in most of the BTIC lines tested, and were absent in pediatric skin-derived precursor cells (SKPs) (Supplementary Figure 5). Sequencing of BTIC lines revealed that the BT147 line encoded a point mutation in HDAC2, and several lines had an increase in HDAC2 copy number variations (CNV) (Supplementary Table 1). Since blood-brain-barrier (BBB) penetration is one of the obstacles impeding successful implementation of HDAC inhibitors in the clinic, we tested an analogue of Entinostat, a known clinical HDAC inhibitor, optimized to improve BBB penetration in a baboon model [37]. This compound (referred to herein as “compound 26”) has been reported to inhibit recombinant human HDAC1 and HDAC2 at nanomolar concentrations [37]. Compound 26 suppressed metabolic activity as determined by alamarBlue assay in a panel of 15 BTIC lines with IC_50_ values ranging from 57 nM to 2735 nM (Figure 5A). BT147 was identified as the most sensitive line, possibly due to the presence of a point mutation in HDAC2 which was also detected in the original patient tumor. Therefore, we focused our studies on the brain-penetrant HDAC inhibitor compound 26.

We first asked whether treatment with compound 26 affected cell viability. BT73 cells were treated with varying concentrations of compound 26 (100-400 nM) and cell viability was determined at 72 and 96 hours post-treatment. The inhibitor significantly decreased cell viability in a dose and time-dependent manner (Figure 5B). To determine whether compound 26 also affected self-renewal, BT73 and BT147 cells were treated with different doses of the inhibitor, and the number of spheres was quantified after 6 days. Compound 26 completely abrogated sphere formation at 5 μM in both BTIC lines (Figure 5C). We next examined the percentage of cells found at the different stages of the cell cycle. BT73 cells were treated with varying concentrations of compound 26 (200 nM-1000 nM) and cellular DNA content examined 72 hours later. The percentage of cells in the G_1_ phase of the cell cycle significantly increased while the percentage in both S and G_2_/M phases was decreased (Figure 5D). Treatment with compound 26 induced hyperacetylation of Histone H3 in a dose-dependent manner, without affecting the total levels of Histone H3, HDAC1 or HDAC2 (Figure 5E). We also observed an increase in the levels of the cyclin-dependent kinase inhibitor p21^CIP1/WAF1^ and cleaved-PARP, suggesting cell cycle arrest and apoptosis as potential mechanisms of action of the inhibitor (Figure 5E). In order to further confirm the mechanism of cell death, we used Annexin V assay to assess the percentage of apoptotic cells (Figure 5F). BT73 cells were treated with varying concentrations of compound 26 (400 nM-1000 nM) and the percentage of both early (Annexin V-positive, PI-negative) and late (Annexin V-positive, PI-positive) apoptotic cells was quantified 72 hours later. The percentage of early and late apoptotic cells significantly increased in a dose-dependent manner as compared to vehicle-treated cells, confirming that compound 26 induced cell death through apoptosis.

### The HDAC inhibitor compound 26 crosses the BBB, accumulates in the brain and induces a significant survival benefit *in vivo* in combination with TMZ

**Figure 6:**
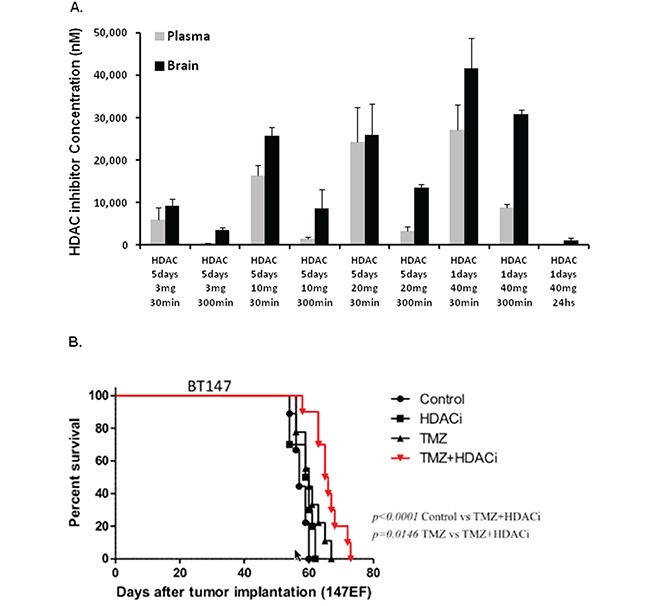
HDAC inhibitor compound 26 crosses the blood brain barrier and extends survival in an orthotopic tumor model in combination with TMZ **A.** Pharmacokinetic results demonstrating plasma and brain concentrations in NOD/SCID mice treated with different doses of compound 26 over 5 days. **B.** NOD/SCID mice bearing intracranial tumors were randomized into 4 groups: 1) vehicle; 2) compound 26, 10 mg/kg, gavage; 3) TMZ, 30 mg/kg, gavage and 4) combination of the 2 compounds. Kaplan-Meyer survival plot is shown. (* p<0.05; ** p<0.01).

Since compound 26 is known to cross the BBB in baboons, we determined the concentration that accumulates in mouse brain and the dose suitable for *in vivo* efficacy studies. A range of doses (3, 10, 20 and 40 mg/kg) was administered *in vivo* over 5 days by oral gavage. Plasma and brains were collected and analyzed by LC/MS. Micromolar concentrations of the drug were detected in both the plasma and the brain (Figure 6A). We chose 10 mg/kg for *in vivo* studies, since the brain concentration achieved at this dose level at 3 hours was 10-fold over the *in vitro* IC_50_ of compound 26 efficacy of seven BTIC lines. To determine *in vivo* efficacy, NOD/SCID mice with small established BT147 intracranial tumors were randomized into 4 groups and treated with 1) vehicle, 2) compound 26 alone (10 mg/kg), 3) TMZ alone (50 mg/kg) or 4) combination of the two drugs for a total of 3 cycles (5 days ON, 2 days OFF). As shown in Figure 6B, treatment with compound 26 alone was ineffective in extending survival. Treatment with TMZ alone had a partial effect, while treatment with both compound 26 and TMZ was able to significantly extend survival as compared to control (approximately 20 days) or single-agent treatment (by approximately 10 days).

### HDAC inhibitor compound 26 is synergistic with UNC1999 *in vitro*

**Figure 7:**
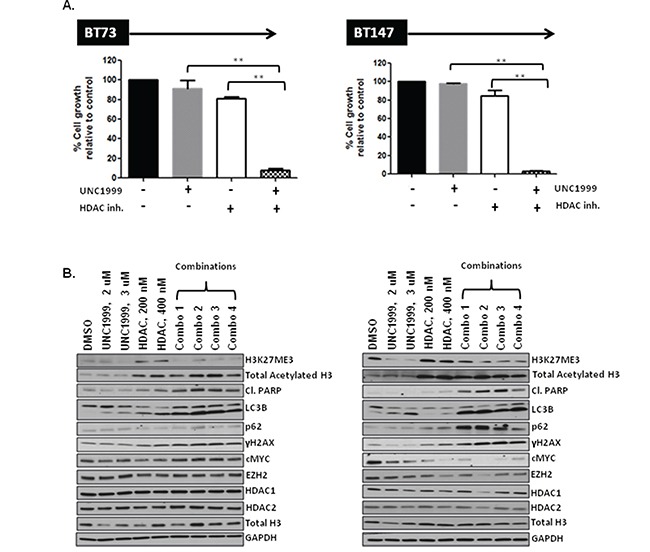
HDAC inhibitor compound 26 synergizes with UNC1999 *in vitro* through augmented apoptosis and DNA damage **A.** Representative bar graphs demonstrating synergy between UNC1999, 3.8 μM and compound 26, 385 nM *in vitro* (n=3; * p<0.05; ** p<0.01). **B.** Representative western blots demonstrating assessment of different targets following treatment with drugs alone and a combination *in vitro.*

Lastly, we examined the efficacy of combining UNC1999 and the HDAC inhibitor. We hypothesized that inhibition of two distinct epigenetic pathways might complement each other and result in strong synergism. Indeed, a combination of UNC1999 with the HDAC inhibitor demonstrated strong synergism in two different BTIC lines *in vitro* with CI values at the ED_50_=0.55, ED_75_=0.49 and ED_90_=0.48 in BT73 and ED_50_=0.35, ED_75_ =0.30 and ED_90_=0.27 in BT147 (Figure 7A). Similarly, we observed either synergistic or additive effects when we tested a combination of compound 26 and two other EZH2 inhibitors, GSK126 and EPZ6438 (Supplementary Figure 6). For GSK126, we observed CI values at the ED_50_, ED_75_, ED_90_ of 1.04, 1.04 and 1.05 in BT73 and ED_50_, ED_75_, ED_90_ of 0.78, 0.70 and 0.64 in BT147. For EPZ6438, we detected CI values at the ED_50_, ED_75_, ED_90_ of 0.6, 0.6 and 0.61 in BT73 and ED_50_, ED_75_, ED_90_ of 0.6, 0.75 and 0.94 in BT147. These results suggest that simultaneously blocking EZH2 and HDAC1/2 may be a potential therapeutic avenue, as we have observed similar synergistic or additive responses with three structurally-different EZH2 inhibitors. We did not observe any additional effect on H3K27me3 or acetylation of Histone H3 with the combination of compound 26 and UNC1999 (Figure 7B), nor did we detect changes in total levels of EZH2, Histone H3, HDAC1 or HDAC2. There was, however, an increase in cleaved-PARP, γH2AX, LC3BII as well as a decrease in c-MYC in cells treated with the combination of drugs, in particular in BT147 cells. These results suggest that several mechanisms are in place that can explain the synergy of the two compounds *in vitro*.

## DISCUSSION

Here we screened a unique epigenetic inhibitor library in order to identify candidate epigenetic modulators that play a role in GBM BTIC growth, survival and self-renewal. To our knowledge, this is the first published report using this collection of drugs in GBM, in particular in primary human BTICs, which are greatly enriched in tumor-initiating capacity as compared to the immortalized cell lines [38]. Our results identify UNC1999 as a potent inhibitor of proliferation of human BTICs *in vitro*. UNC1999 is a novel SAM-competitive EZH2/EZH1 inhibitor that has demonstrated efficacy in several cancer models such as leukemia, multiple myeloma and colon cancer [29, 39, 40]. We show here that UNC1999 is cytotoxic *in vitro* in a diverse panel of GBM BTIC lines, inhibits self-renewal, causes cell cycle arrest at G1 stage and induces down-regulation of H3K27me3. Our data suggests that UNC1999-induced cell death is independent of either apoptosis or necroptosis, and may induce defective autophagy, as judged by the accumulation of both LC3BII and p62/SQSTM1. A similar mechanism of action has been recently described by Button et al. for dual PI3K/mTOR inhibitors [35].

Increased and decreased expression of EZH2 have both been implicated in the development and progression of a variety of cancers types. EZH2 overexpression is associated with aggressiveness and worse prognosis in prostate, breast, bladder, endometrial tumors, melanoma and GBM [30]. Additionally, gain-of-function point mutations at Y641 of EZH2 were identified in 22% of DLBCL and 7-12% of follicular lymphomas [41]. These mutations result in enhanced enzymatic function of EZH2 leading to increased levels of tri-methylated H3K27 and repression of PRC2 targets. However, EZH2 can also act as a tumor suppressor, with loss-of-function mutations identified in a subset of myelodisplastic syndromes, myeloproliferative neoplasms and T cell acute lymphoblastic leukemias. Moreover, recurrent missense mutations in the PRC2 substrate lysine residue 27 of histone H3 and its variants occur in 31% of pediatric GBM, 78% of diffuse intrinsic pontine glioma (DIPG) and 50% of pediatric high-grade gliomas (pHGG), but not in adult GBM. The H3K27M mutation is oncogenic, blocking growth-suppressive PRC2 methylation activity resulting in reduced H3K27 methylation and derepression of PRC2 target genes. In this regard, inhibition of H3K27 demethylation mediated by JMJD3, using the inhibitor GSK-J4, suppressed the growth of DIPG tumors grown as xenografts [24]. Thus, while EZH2 inhibition may prove beneficial in adult GBM, it will most likely be detrimental for patients with pediatric gliomas.

Until now, most of the studies in GBM have used either EZH2 knockdown or treatment with 3-deazaneplanocin (DZNep) in order to assess the effect of EZH2 inhibition [19, 42, 43]. However, DZNep is a potent S-adenosylhomocysteine inhibitor which inhibits EZH2 in an indirect manner though depletion of cellular levels of all PRC2 components, resulting in decreased H3K27 methylation. In contrast, UNC1999 directly inhibits EZH2 and in a potent and selective manner. Interestingly, de Vries et al. reported that prolonged EZH2 depletion in glioma switched tumor cells to a different epigenetic state that enhanced cell proliferation and DNA damage repair, resulting in tumor progression [44]. However, short-term EZH2 depletion significantly improved survival, suggesting that determining the precise dosing schedules will be crucial for a successful implementation of EZH2 inhibitors into the clinic.

We hypothesize that the superior efficacy of UNC1999 *in vitro* as compared to other EZH2 inhibitors may be due to its ability to simultaneously target both EZH2 and EZH1. Konze et al. [31], which first characterized UNC1999, found that it was 10,000-fold more selective for EZH2 as compared to other methyltransferases (Konze et al., Figure 2G). The only other histone methyl transferase that UNC1999 inhibited was EZH1, albeit 10-fold less potently than EZH2. In contrast, GSK126 and EPZ-6438 were over 50-fold more potent towards EZH1 than EZH2. This finding suggests that UNC1999 may be more effective in inducing BTIC cell death due to suppression of both EZH2 and EZH1.

An additional issue regarding the existing EZH2 and HDAC class I inhibitors is their inability to penetrate the blood-brain barrier (BBB) [45]. BBB penetration is one of the major issues impeding successful therapeutic targeting in GBM, as more than 98% of drugs fail to cross the BBB. Although UNC1999 did not cross the BBB, we report on the efficacy of a promising class I HDAC inhibitor, optimized for optimal brain penetration. This inhibitor was identified when a series of compounds using Entinostat as a backbone were synthesized and tested in baboons [37]. Compound 26 from these studies potently inhibited HDAC1 and HDAC2 and crossed the BBB. This inhibitor exhibited nanomolar to low micromolar cytotoxicity *in vitro*, disrupted self-renewal, caused cell cycle arrest, induced apoptosis and hyper-acetylation of histone H3 and demonstrated efficacy in an orthotopic BTIC model in combination with TMZ. Interestingly, we have also found that a combination of the UNC1999 EZH2 and compound 26 HDAC class I inhibitors demonstrated synergy *in vitro*. This combination has been previously tested in other cancers such as gallbladder carcinoma [46], however we believe this is the first report that demonstrates such synergy in GBM.

## MATERIALS AND METHODS

### Primary cells

GBM BTIC lines BT50, BT53, BT67, BT73, BT75, BT92, BT100, BT119 BT143 and BT147 were previously described [14–16, 18]. BTIC lines BT69, BT140, BT161, BT167, BT194, BT198 and BT208 were cultured from a series of tumor specimens (see Supplementary Table 2) obtained following informed consent from adult GBM patients during their operative procedure as previously described [14] with the approval of the University of Calgary Ethics Review Board. Briefly, BTIC cultures were initiated in defined culture serum-free medium (SFM) and gave rise to non-adherent spheres after 7–21 days in culture. Primary tumor spheres were expanded for several passages and then cryopreserved in 10% DMSO in SFM until use in experiments (14). All BTIC lines were used at passages of approximately 10-20.

### Epigenetic library

The epigenetic library consists of 24 small molecule compounds targeting diverse epigenetic targets. The library (http://www.thesgc.org/chemical-probes) was kindly provided by the Structural Genomics Consortium (SGC, Toronto, ON; http://www.thesgc.org).

### Drugs

UNC1999, GSK343, EPZ6438, GSK126 and Necrostatin were from Selleck Chemicals, Dexamethasone and Temozolomide from Sigma, Z-VAD-FMK from Calbiochem, and UNC2400, a negative control for UNC1999, was kindly provided by the SGC. The brain penetrant HDAC inhibitor Compound 26 was obtained from a six step synthesis starting from 4-bromo-2-nitroaniline based on a previously reported synthetic route [37]; dx.doi.org/10.1021/cn500021p).

### Primary screening assay

Five BTIC lines (BT143, BT53, BT69, BT100 and BT194) were dissociated into single cells and seeded at 1000-2500 cells/well in 50 μL medium in 384-well microplates. Compounds were dissolved in DMSO as 10 mM stocks. Compound addition was performed at OICR using HP Tecan D300 digital dispenser. For each compound, 12-point, 2-fold serial dilutions were performed. Drug effects were compared to cells optimally proliferating in 0.1% DMSO alone, while wells filled with media served as background. AlamarBlue® (5 μL) was added after 3 or 6 days, and fluorescence intensity measured after 6 hr on a PHERAstar microplate reader, equipped with a λ540 excitation/ λ590 emission filter.

### Whole genome and transcriptome sequencing

DNA and RNA from each BTIC line and DNA from peripheral blood from the respective patients were used to construct genome and transcriptome libraries. Whole genome and transcriptome paired-end sequencing using the Illumina HiSeq2500 platform generated an average genome coverage of 40X and average transcriptome read counts of 200 million. Genomic sequence data from normal and tumor were then aligned to the human reference genome build GRCh37 using BWA (v0.5.7). Data from multiple lanes were merged and duplicates identified using Picard (v1.38). CNASeq (v0.0.6) and APOLLOH (v0.1.1) were used to identify copy number aberrations and loss of heterozygosity respectively. For identification of single nucleotide variants, Samtools (v1.0.2) was applied followed by filtering with MutationSeq (v1.0.2), and the results were combined with variant called with Strelka (v0.4.6.2). Small indels were also identified using Strelka. These variants were annotated using Ensembl (v69). Transcriptome reads were gap-aligned using the Jaguar pipeline (v2.0.3). Further in-house tools were used to generate read counts for exons and genes and calculate expression levels using reads per kilobase per million mapped reads (RPKM).

### Western blot analysis

For cell lysis, cells were collected, washed in cold PBS, and lysates prepared in RIPA lysis buffer. Equal amounts of protein were resolved on gradient polyacrylamide gels and subjected to immunoblotting with the following antibodies: rabbit anti-EZH2 (1:1000, CST#5246), rabbit anti-H3K27me3 (1:1000, CST#9733), rabbit anti-H3K27me2 (1:1000, CST#9755), rabbit anti-H3K4me1 (1:1000, CST#5326), rabbit anti-H3K9me2 (1:1000, CST#4658), mouse anti-H3K27ac (1:2500, Milllipore 17-683), rabbit anti-histoneH3 (1:1000, Millipore 05-928), rabbit anti-cleaved PARP (1:1000, CST#9541L), rabbit anti-LC3B (1:1000, CST#3868P), rabbit anti-cMYC (1:1000, CST#13987), rabbit anti-acetyl histone H3 (1:1000, Millipore 06-599), rabbit anti-p21 (1:1000, CST#2947S), mouse anti-HDAC1 (1:1000, CST#5356), mouse anti-HDAC2 (1:1000, CST#5113), rabbit anti-SQSTM1/p62 (1:1000, CST#8025), rabbit anti-phospho-Histone H2A. X (1:1000, Millipore 05-636), and rabbit anti-GAPDH (1:1000, CST#5174). HRP-conjugated goat anti-mouse IgG (1:5000) and goat anti-rabbit IgG (1:10,000) were used as the secondary antibodies.

### Immunofluorescence

Cells underwent Cytospin, fixed either with 4% PFA (anti-SQSTM1/p62) or 100% methanol (anti-LC3B), permeabilized with PBS/0.2% Triton X-100, washed with PBS/ 100 mM Glycine and then incubated in IF buffer (PBS, 0.1% BSA, 0.2% Triton X-100, 0.05% Tween-20), with 2% BSA for 30 minutes as a blocking solution. Slides with cells were incubated with primary antibodies in blocking solution at 4°C overnight. After washing with IF buffer, the cells were incubated with secondary antibodies in blocking solution for 1 hr at RT. Finally, after PBS washes, slides were mounted in Permount solution (Thermo, Walthman, MA). Digital image acquisition was performed with Axiovision software (Zeiss, Oberkochen, Germany) on a Zeiss Axioplan 2 microscope with a Hamamatsu (Bridgewater, NJ) Orca-R2 CCD video camera.

### Drug combination studies

The combination index (CI) was used to evaluate the interaction between different drugs [47]. BT73 and BT147 were dissociated into single cells, seeded at 1000-3000 cells/well in 100 μL medium in 96-well plates and treated with increasing concentrations of UNC1999, HDAC inhibitor, DEX and TMZ. Six days after the incubation, alamarBlue® (10 μL) was added to the plates and fluorescence intensity measured after 6 hr on a PHERAstar microplate reader, equipped with a λ540 excitation/λ590 emission filter. The CalcuSyn median effect model was used to calculate the CI values and evaluate whether the drug combinations were synergistic, antagonistic, or additive. CI values of < 1 indicate synergism, CI = 1 indicate additivity, and CI > 1 indicate antagonism [47].

### Pharmacokinetic (PK) properties and blood brain barrier (BBB) penetration of compounds

To obtain plasma exposure and BBB penetration of compounds at various time points, blood and brains were collected form mouse after dosing compounds at different time points. Plasma was separated from blood by centrifugation. For analysis 20 μL plasma was transferred into a 1.5 mL microcentrifuge tube, to which 40 μL acetonitrile was added to denature and precipitate proteins, and were centrifuged for 6.5 min. at 14000 rpm. The supernatant was transferred to LCMS vials for analysis.

For quantification of drug levels in brain samples, brains were transferred to plastic vials containing 1:2 (brain:water, e.g. 300 mg brain with 600 μL water) volume of water and 2 glass Pyrex beads (4mm in size). Samples were homogenized by bead beating (3 × 15 second pulses of 5,000 rpm) using a Precellys 24 homogenizer (Bertin Technologies, Montigny-le-Bretonneux, France). Each sample was mixed vigorously with 2 volumes of acetonitrile to precipitate out protein and were centrifuged for 6.5 min. at 14000 rpm. The supernatant was then transferred to LCMS vials for analysis. The concentration in brain was corrected for dilution.

### LCMS analysis

For each analysis, a calibration curve with ten different concentrations starting from 10 mg/mL up to 10,000 ng/mL was prepared by spiking blank plasma. Chromatographic separations were carried out on an Acquity UPLC BEH C18 (2.1 × 50 mm, 1.7 μm) column using ACQUITY UPLC II system. The mobile phase was 0.1% formic acid in water (solvent A) and 0.1% formic acid in acetonitrile (solvent B). A gradient starting at 95% solvent A going to 5% in 4.5 min., holding for 0.5 min., going back to 95% in 0.5 min. and equilibrating the column for 1 min. was employed. A Waters Synapt G2S QTof mass spectrometer equipped with an atmospheric pressure ionization source was used for mass spectrometric analysis. MassLynx 4.1 was used for data analysis.

### Determination of cellular DNA content

The BTIC line BT73 (0.2×10^6^) was plated in 6-well dishes and DMSO, UNC1999 (2 μM, 3 μM and 5 μM) or HDAC inhibitor (0.2 μM, 0.4 μM, 0.6 μM and 1 μM) added. After 48 hrs (UNC1999) or 72 hrs (HDAC inhibitor), cells were harvested, washed with PBS and fixed in 70% ice-cold ethanol. Fixed cells were treated with RNAseA, stained with Propidium Iodide at 37°C for 1 hr and analyzed on a LSR II flow cytometer to determine cellular DNA content.

### Viable cell counts following treatment with UNC1999 and HDAC inhibitor

1×10^5^ dissociated BTICs (BT73) were seeded in triplicate in 12-well non-tissue culture-treated plates in 2 mL of medium. UNC1999 (4 μM - 6 μM), HDAC inhibitor (0.1 μM - 0.4 μM) or 0.1% DMSO were added to the cells 24 hrs later. At specific time points, spheres were collected, dissociated and subjected to a viable cell count by trypan blue exclusion.

### Annexin V assay

The ability of drugs to induce apoptosis in BTICs was determined with an Annexin V-FITC detection kit, used according to the manufacturer's instructions (BD Pharmingen). Briefly, 0.3×10^6^ BTICs (BT73) were plated in 6-well dishes and treated with various concentrations of UNC1999 (3-6 μM) or HDAC inhibitor (0.4 – 1 μM). Cells were harvested 24-72 hr later, stained for Annexin V/propidium iodide and analyzed on a LSR II flow cytometer. Relative numbers of early apoptotic cells (Annexin V-positive, Propidium iodide-negative) and late apoptotic cells (Annexin V-positive, Propidium iodide-positive) were obtained for each time point.

### Sphere-formation assay

BTICs (BT73 and BT147) were seeded in triplicate in non-TC-treated 96-well microplates at a density of 1000-2500 cells/well in 50 μL/well. Compounds were diluted in medium (1:1000) and immediately added to the cells in a volume of 50 μL (final concentration of DMSO = 0.05%). Cells were re-treated 72 hrs post-plating with drugs, and fixed after 6 days with 4% paraformaldehyde (Electron Microscopy Sciences). Sphere number was determined by manual counting, and the results expressed as the mean sphere number of treated wells as compared to DMSO-treated wells*100.

### Flank xenograft model

1.5×10^6^ BTICs (73M) were resuspended in media and injected in 100 μl volume subcutaneously into 6-8-week old NOD/SCID mice. Drug treatment began when tumor size reached ~25 mm^3^. Animals were randomized into 4 groups: 1) Vehicle; 2) UNC1999 alone; 3) DEX alone; 4) UNC1999 + DEX. Mice were injected via oral gavage with either vehicle (10% DMSO, 40% PEG) or UNC1999 (150 mg/kg) every day for 17 days. An additional group of mice received intraperitoneal injection of DEX (1 mg/kg) every day for 17 days. The combination group received both UNC1999 and DEX administered by oral gavage and i.p. injection respectively.

### Orthotopic xenograft model

BT147 spheres were dissociated into single cell suspensions and 1×10^5^ cells were stereotactically implanted into the right striata of 6-8 week old NOD/SCID mice as previously described [14]. Seven days after BTIC implantation, mice were randomized into 4 groups and injected via oral gavage for a total of 3 cycles (5 days ON, 2 days OFF): 1) vehicle (50% PEG, 50% water); 2) HDAC inhibitor alone (10 mg/kg); 3) Temozolomide (TMZ) alone (30 mg/kg) and 4) combination of HDAC inhibitor and Temozolomide.

### Statistical analysis

Statistical analyses were performed with Microsoft Excel using an unpaired, two-tailed student's T-test with P<0.05 as the significance cutoff. One-way ANOVA was used to determine statistically significant differences from the mean in the combination study *in vivo*.

## SUPPLEMENTARY FIGURES AND TABLES



## Abbreviations

GBM: Glioblastoma
BTICs: Brain tumor-initiating cells
SGC: Structural Genomics Consortium
BBB: Blood-brain-barrier
TMZ: Temozolomide
MGMT: O-6-methylguanine-DNA methyltransferase
DEX: Dexamethasone
NOD/SCID: Non-obese diabetic/severe combined immune deficiency
SKPs: Skin-derived precursor cells EZH2 Enhancer of zeste homologue 2
HDAC: Histone deacetylase
SAM: S-adenosyl methionine
PRC2: Polycomb repressive complex 2
CI: combination index
DZNep: 3-deazaneplanocin
