# Supplementary material for: Small molecule epigenetic screen identifies novel EZH2 and HDAC inhibitors that target glioblastoma brain tumor-initiating cells
Source: Oncotarget. 2016 Jul 18;7(37):59360–76. doi: 10.18632/oncotarget.10661 (PMC5312317; doi:10.18632/oncotarget.10661)
Supplement: Supplementary file 1 [file oncotarget-07-59360-s001.pdf]

Small molecule epigenetic screen identifies novel EZH2 and HDAC inhibitors that target glioblastoma brain tumor-initiating cells

SUPPLEMENTARY FIGURES AND TABLES

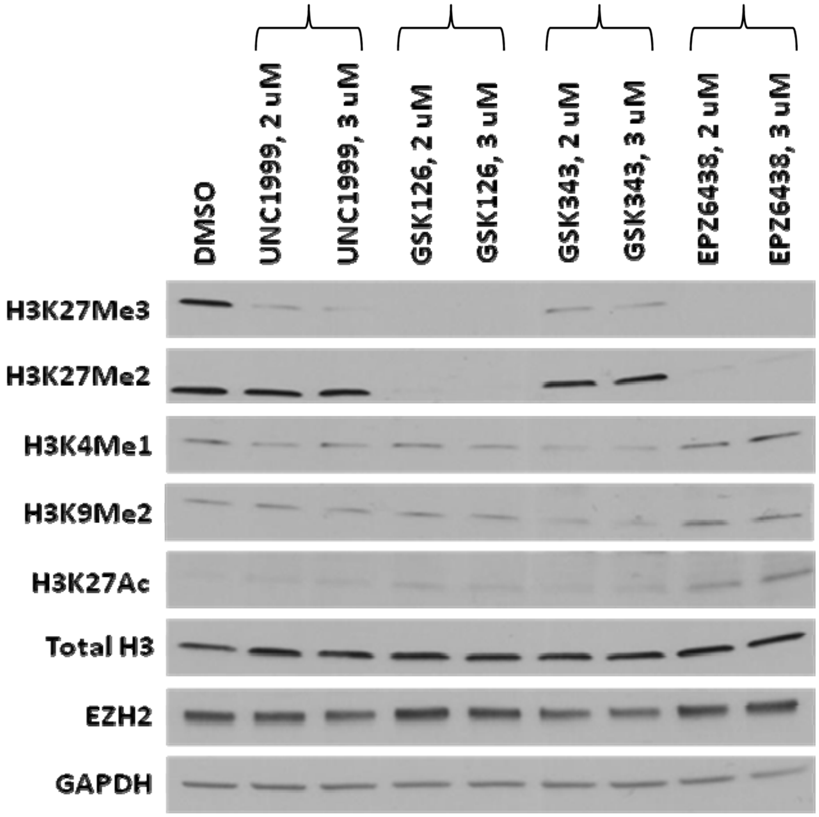

Supplementary Figure 1: UNC1999 inhibits only trimethylation of H3K27. Representative western blot demonstrates the effect of treatment with UNC1999 and other EZH2 inhibitors (GSK343, GSK126 EPZ6438) on H3K27Me3, H3K27Me2, H3K4Me1, H3K9Me2, H3K27Ac, total Histone H3 and total EZH2 in BT73.

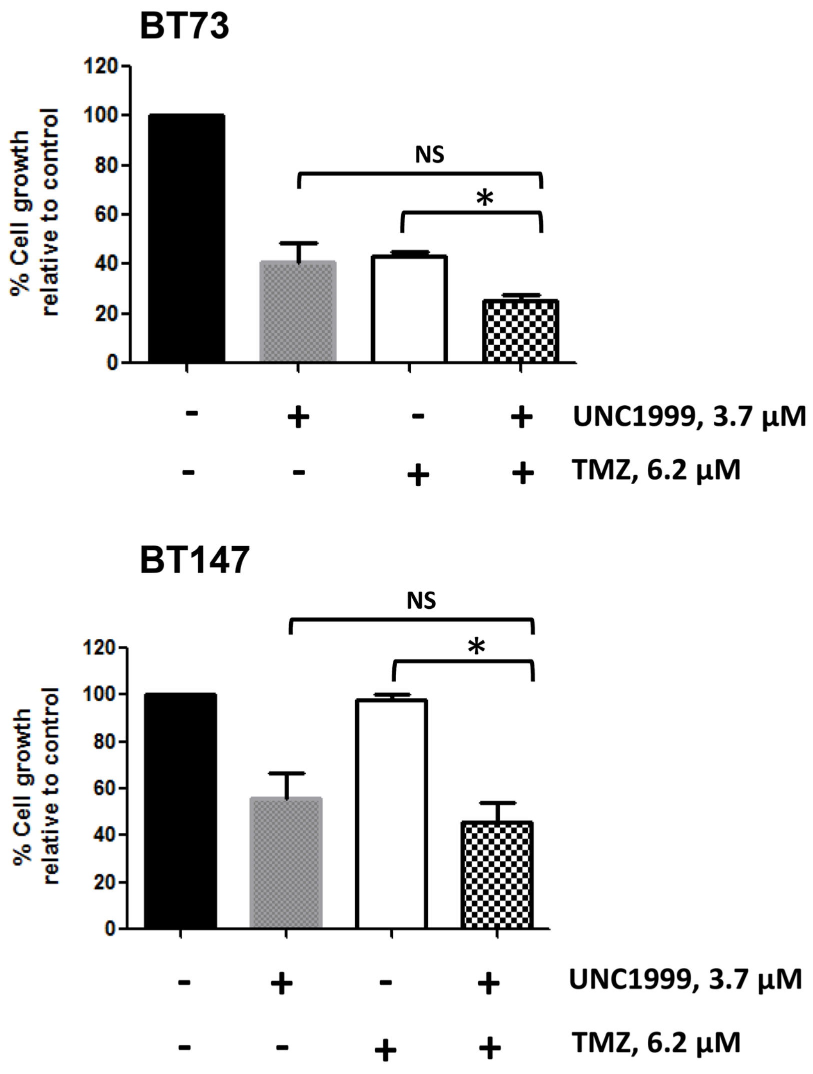

Supplementary Figure 2: UNC1999 does not synergize with TMZ *in vitro*. Representative bar graphs demonstrating the lack of synergy between UNC1999, 3.7 µM and TMZ, 6.2 µM are shown.

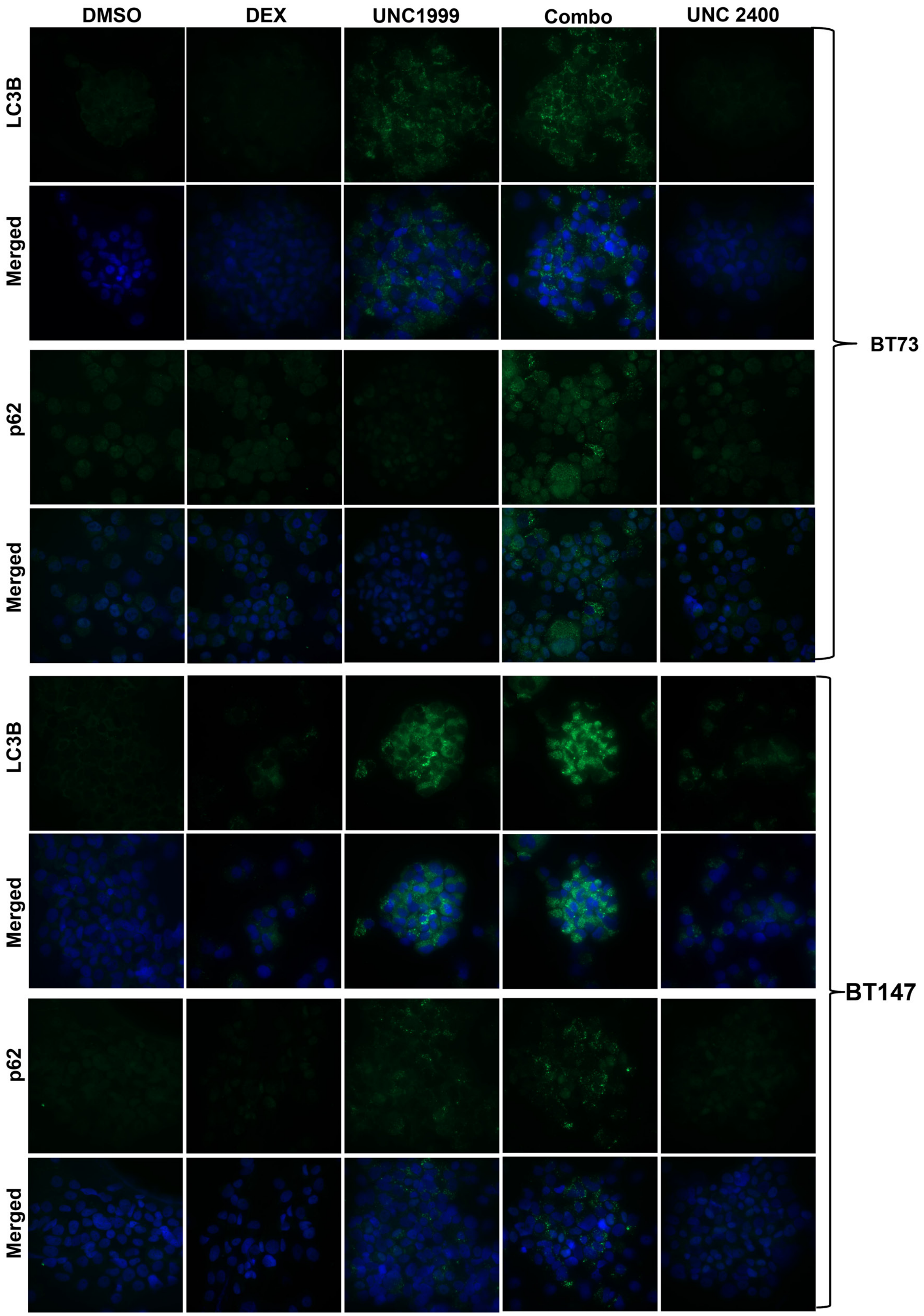

Supplementary Figure 3: Treatment with UNC1999 and DEX induced the accumulation of p62/SQTM1. BT73 and BT147 were treated with UNC1999 and DEX alone and in combination for 72 hours, underwent Cytospin, were fixed and stained for LC3B and p62. Digital image acquisition was performed on a Zeiss Axioplan 2 microscope with a Hamamatsu (Bridgewater, NJ) Orca-R2 CCD video camera.

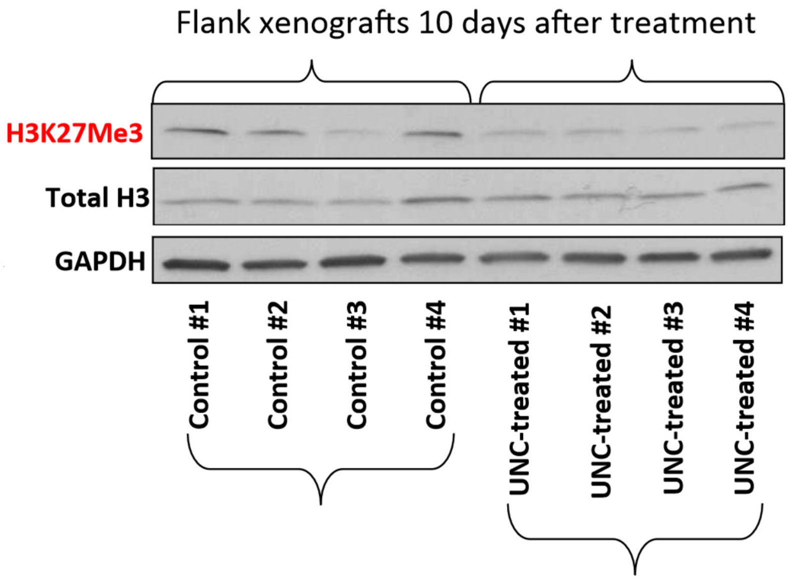

Supplementary Figure 4: UNC1999 decreases H3K27Me3 *in vivo*. 1.5x10^6 BTICs (73M) were resuspended in media and injected in 100 µl volume subcutaneously into 6-8-week old NOD/SCID mice. Drug treatment began when tumor size reached ~25 mm^3. Mice were injected with either vehicle (10% DMSO, 40% PEG in water) or UNC1999 (150 mg/kg) every day for 10 days. At the end of the treament, tumors were extracted, lysed and target inhibition determined by immunoblotting.

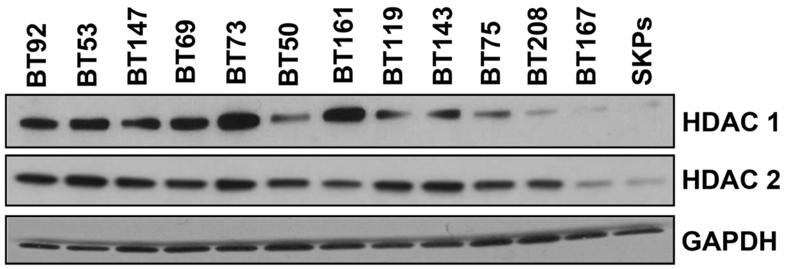

Supplementary Figure 5: HDAC1 and HDAC2 protein expression in BTICs and SKPs as assessed by immunoblotting.

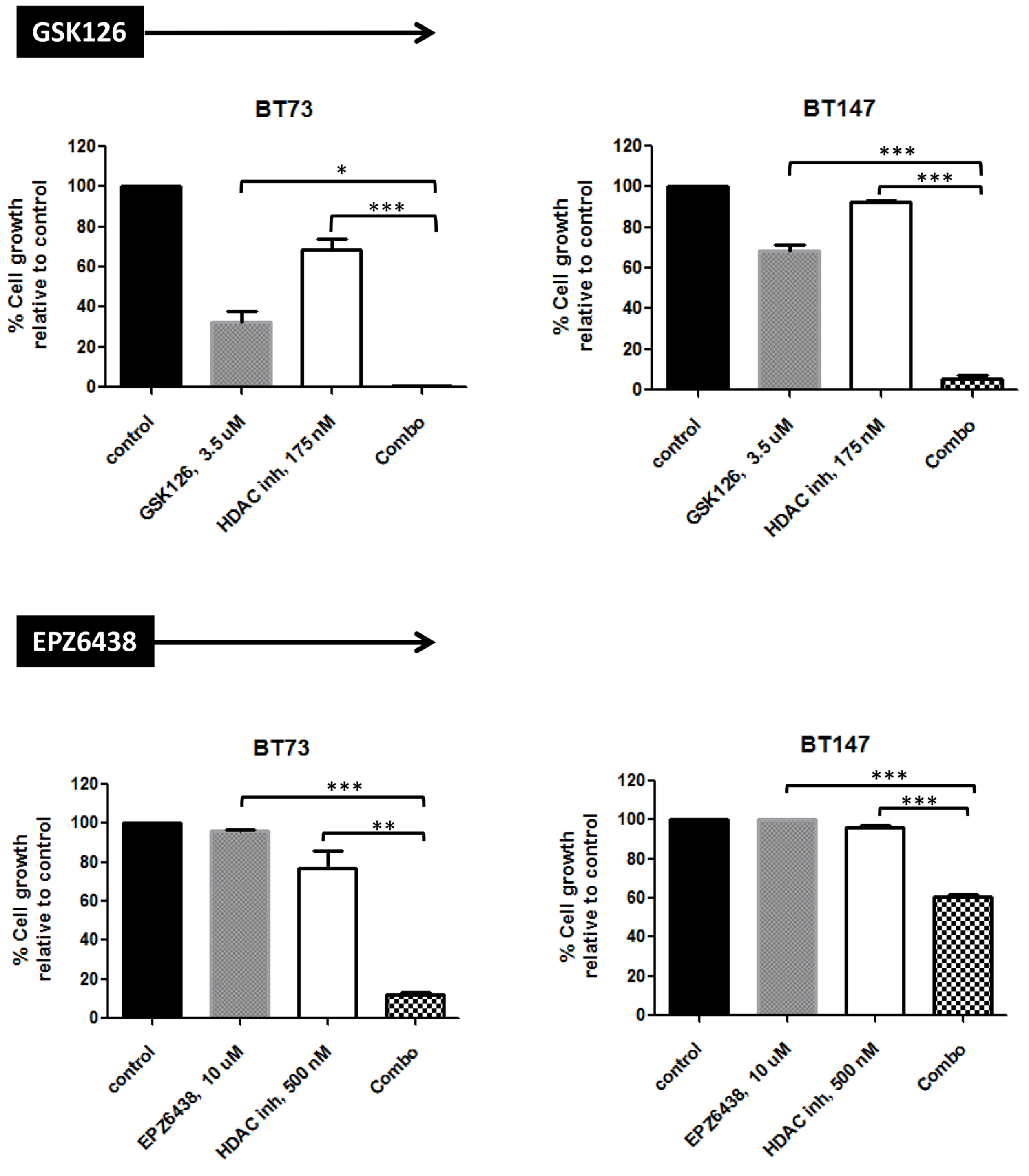

Supplementary Figure 6: Combination of HDAC inhibitor compound 26 with EZH2 inhibitors GSK126 and EPZ6438 demonstrates synergy and additivity *in vitro*. Representative bar graphs demonstrating combination efficacy are shown.

Supplementary Table 1: BTIC lines information, including SNV, CNV and expression data for EZH2, HDAC1 and HDAC2

BTIC ID	SNV_EZH2	CNV_ EZH2	EXPR_ EZH2	SNV_ HDAC1	CNV_ HDAC1	EXPR_ HDAC1	SNV_HDAC2	CNV_ HDAC2	EXPR_ HDAC2
**BT-92**	WT	3.36	33.00	WT	3	6.68	WT	1.75	7.51
**BT-53**	WT	3	21.84	WT	3	16.81	WT	2	12.05
**BT-147**	WT	3	15.70	WT	2	12.64	1NON_ SYNONYMOUS_ CODING	2	6.96
**BT-69**	WT	3	11.82	WT	2	9.16	WT	2	7.93
**BT-73**	WT	4	11.57	WT	3	22.16	WT	2	9.90
**BT-50**	WT	3	11.68	WT	2	8.70	WT	2	5.82
**BT-161**	WT	3	11.47	WT	2.59	10.71	WT	1	4.93
**BT-119**	WT	3	9.65	WT	3	9.97	WT	2	9.18
**BT-143**	2NON_ SYNONYMOUS_ CODING	2	5.11	WT	2	10.34	WT	2	5.87
**BT-75**	WT	3	4.28	WT	2	8.11	WT	2	6.13
**BT-208**	WT	3	2.27	WT	2	9.49	WT	1	2.56
**BT-167**	WT	2	2.15	WT	3	12.75	WT	1	1.83

Red font highlights mutations in either EGFR, PTEN or p53.

Supplementary Table 2: GBM patient information, including age at diagnosis, treatment, MGMT methylation status and mutational status of EGFR, PTEN, p53 and IDH1

BTIC ID	Age at diagnosis	Sex	Patient Dx	New or Reccurent	Initial Rx CRT/ RT/OT	Patient Survival (days)	Patient Mol. Subtype	M/U	BTIC EGFR Status	BTIC p53 Status	BTIC PTEN status	BTIC IDH1 status
BT-50	62	M	GBM	N	No treatment	108	N/A	M	WT	WT	HET	WT
BT-53	N/A	M	GBM	N	Unknown	N/A	PRO	M	MT/ VIII	MT	WT	WT
BT-67	44	M	GBM	N	RT+TMZ	82	MES/ CLAS	M	WT	WT	HET	WT
BT-69	51	M	GBM	N	No treatment	108	CLAS	M	MT	WT	MT	WT
BT-73	52	M	GBM	N	Unknown	91	CLAS	M	MT	MT	MT	WT
BT-75	74	M	GBM	N	RT alone	200	PRO	U	WT	WT	WT	WT
BT-100	63	M	GBM	N	No treatment	40	MES	M	WT	WT	MT	WT
BT-92	23	M	GBM	R	RT+TMZ followed by TMZ	561	PRO	U	MT	MT	MT	WT
BT-119	69	F	GBM	R	RT+TMZ followed by TMZ	558	CLAS	M	MT	MT	HET/ HET	WT
BT-140	63	M	GBM	N	RT+TMZ followed by TMZ	192	PRO	U	MT	WT	MT	WT
BT-143	39	F	GBM	R	RT+TMZ followed by TMZ	2751	CLAS	M	MT	MT	HET	WT
BT-147	55	M	GBM	R	RT+TMZ followed by TMZ	635	CLAS	U	VIII	MT	MT	WT
BT-161	55	F	GBM	N	RT+TMZ+placebo or bevacizumab followed by TMZ	1028	CLAS	M	WT	WT	MT	WT
BT-167	63	M	GBM	R	RT+TMZ followed by TMZ	459	MES	N/D	WT	WT	MT	WT
BT-194	59	M	GBM	N	CRT (Stupp), then RESCUE after progression/ recurrence	1261	CLAS	M	WT	WT	WT	WT
BT-198	52	F	GBM	N	No treatment	101	PRO	M	WT	MT	MT	WT
BT-208	69	M	GBM	N	RT+TMZ followed by TMZ	600	CLAS	M	WT	WT	MT	WT
BT-280	35	M	GBM	R	RT alone (CEC1 trial), then RESCUE after recurrence	351	CLAS	N/A	WT	MT	WT	WT

Red font highlights mutations in either HDAC2 or EZH2.
